# Hepatocellular Carcinoma and Sex Hormones

**DOI:** 10.1155/1992/72761

**Published:** 1992

**Authors:** Naofumi Nagasue, Hitoshi Kohno

**Affiliations:** Second Department of Surgery Shimane Medical University Izumo 693 Japan

## Abstract

The liver is morphologically and functionally modulated by sex hormones. Long-term use of oral contraceptives and androgenic steroids can induce benign and malignant hepatocellular tumors. Hepatocellular carcinoma (HCC) is more prevalent in men than in women. The role of sex hormones and their receptors in the development of HCC was reviewed. Some HCCs may be androgen dependent but others may be estrogen or even both dependent. Further studies are mandatory in order to utilize such characteristics of HCC for an effective prophylaxis and therapy of this tumor.


*HPB Surgery*, 1992, Vol. 6, pp. 1–6
Reprints available directly from the publisher
Photocopying permitted by license only

© 1992 Harwood Academic Publishers GmbH
Printed in the United Kingdom

### REVIEW ARTICLE

### HEPATOCELLULAR CARCINOMA AND SEX HORMONES

NAOFUMI NAGASUE and HITOSHI KOHNO
*Second Department of Surgery, Shimane Medical University, Izumo 693, Japan*

*(Received 5 December 1991)*

The liver is morphologically and functionally modulated by sex hormones. Long-term use of oral contraceptives and androgenic steroids can induce benign and malignant hepatocellular tumors. Hepatocellular carcinoma (HCC) is more prevalent in men than in women. The role of sex hormones and their receptors in the development of HCC was reviewed. Some HCCs may be androgen dependent but others may be estrogen or even both dependent. Further studies are mandatory in order to utilize such characteristics of HCC for an effective prophylaxis and therapy of this tumor.

KEY WORDS: Hepatocellular carcinoma, sex hormone, androgen, estrogen, progesterone, receptor

Cirrhosis of the liver is frequently associated with hepatocellular carcinoma (HCC). It is well known that cirrhotic male patients, particularly those with alcoholic cirrhosis, have impaired function of the hypothalamic-pituitary-gonadal axis[1]. All over the world, HCC is more prevalent in men than in women. This is especially true in patients with cirrhosis and in the areas where HCC is common. Such male predominance of HCC may be related to sex hormone imbalance[2]. On the other hand, it is undeniable that long-term use of oral contraceptives induces not only benign but malignant tumors of the liver. Also, androgenic anabolic steroids, especially 17-alkyl-substituted steroids, seem to be related to the development of liver neoplasms including HCC. These facts raise the question; what is the genuine role of sex hormones in hepatocarcinogenesis in experimental animals and particularly in humans?

### *Liver Tumors and Sex Steroid Administration*

The effect of sex hormones on liver function and morphologic features has been pointed out over the past two decades. It is generally accepted that the liver is one of the target organs for estrogens. Since the first report by Baum *et al.*[3], a possible correlation of estrogens with the development of hepatic neoplasms has been suggested by numerous reports of oral contraceptive-associated focal nodular hyperplasia (FNH) or liver cell adenoma. Malignant transformation has also been reported in such patients. In addition, withdrawal of oral contraceptives occasionally induces regression of the drug induced hepatic tumors[4–6]. According to the recent articles by Neuberger *et al.* and Forman *et al*[8]., the use of contraceptive pills for longer than 8 years is associated with a 20-fold increased risk of HCC.

On the other hand, the administration of exogenous androgens, particularly 17-$\alpha$-alkylated androgens, causes various hepatic lesions such as dilation of biliary canaliculi, loss of canalicular villi, peliosis hepatis, adenoma, and HCC[9]. Such changes occur less frequently with non-17-$\alpha$-alkylated androgens. It is noteworthy that synthetic estrogen and progestogen used for pills have a similar chemical structure to androgenic steroids.

Although most carcinogens can be shown to be mutagenic *in vitro*, this has not been demonstrated for estrogens[10]. The Committee on Safety of Medicine (London) reported a tumorigenic action of synthetic estrogens in male rats but not in female rats[11]. Reznik-Schuller[12] found the development of liver tumors (hepatocellular adenomas, carcinomas, and cholangiocarcinoma) in 29% of male European hamsters treated with diethylstilbestrol (DES). This seems to be the first report showing that estrogens alone can be carcinogenic. Other authors have suggested that estrogens promote the effect of carcinogens[13–15]. There are several groups in Japan who have extensively studied this problem. Sumi *et al.*[16] initially found a synergistic effect of DES and carcinogens on the development of liver tumors in male WF rats. Later, they have found that DES itself has a direct carcinogenic effect on the liver and that this effect is not mediated by prolactin[17]. Higashi *et al.*[18] in 1980 reported that synthetic estrogen and progestogen could induce HCC in female Wistar rats.

Despite a considerable number of reports on androgenic steroid-induced hepatic tumors, there is limited evidence for a direct etiologic relationship between administration of the drugs and HCC in humans[19]. Many years ago, Morris *et al.*[20] found that in a hepatic carcinogenesis model in rats with 2-diacetylaminofluorene HCC occurs more frequently in intact males and castrated females treated with testosterone than in intact females, castrated males, castrated females, and castrated males treated with DES. Whether or not androgenic steroids are carcinogenic remains to be elucidated.

### *Sex Hormone Receptors in HCC*

It is theoretically assumed that the tumorigenic effect of estrogens may be through an estrogen receptor (ER). The presence of ER in human liver was first found by Duffy and Duffy[21] in 1978. Friedman *et al.*[22] estimated ER levels in human HCCs. The titer of ER in HCC taken from 5 male adults ranged from 0.9 to 6.4 fmol/mg cytosol protein. We have measured ER of HCC in 30 adult patients[23]. ERs were present in 12 (40%) tumors. Since 29 were male in that study, we next assayed the receptor in 19 women[24]. ERs were detected in 7 of them (37%). Ohnishi *et al.*[25] found the receptor in only one of 6 HCCs. At present, it is not well known if the presence of ER in HCC is really related to the development and growth of the tumor in humans. Li and Li[26] suggested a possibly important role of ER in hepatocarcinogenesis since a higher value of both cytosolic and nuclear ERs in hamster livers was observed when the regimen of DES and $\alpha$-naphthoflavon was made more tumorigenic. Considering this and previously described experimental and clinical studies, ERs in the liver or HCC may also play a role in human hepatic carcinogenesis.

It has long been believed that human liver is not a target organ for androgen. In

1985, we first observed that normal human liver possesses androgen receptors (AR)[27]. Immediately after our report, Bannister *et al.*[28] presented a similar observation. Iqbal *et al.*[29] were the first to find ARs in human HCC. We have also shown that ARs are present in 74% of male and 37% of female HCCs[24,27]. In addition, the titers were significantly higher in HCC than in the surrounding liver parenchyma. A very similar result was presented by Ohnishi *et al.*[25]. We thereafter investigated, with positive results, if extrincically given testosterones were taken up by AR in HCC using autoradiographic techniques. All over the world, HCC is more prevalent in males than it is in females[31]. This is especially true in patients with liver cirrhosis[32]. Male predominance is usually explained by the fact that alcoholism, chronic liver disease, and in particular chronic hepatitis B infection are more prevalent among males. In the light of our serial receptor studies, however, we have suggested that some, if not all, human HCC may be androgen-dependent. Such a speculation is supported by our clinical results which will be described in the next section.

Several investigative groups have shown that high titers of progesterone receptor (PgR) are present in FNH and liver cell adenoma whether or not they are induced by oral contraceptives[33-35]. Demenes *et al.*[36] found a similar result in hepatoblastoma. However, very little is known of whether PgR is present in HCC or not[22,34]. We estimated PgR as well as AR and ER in 22 consecutive patients with HCC[37]. PgR was considered to exist when the titer was higher than 1.0 fmol/mg of protein. The receptor was detected in the cytosol of HCC in 4 patients (18.2%), and the titers were low ranging from 1.1 to 3.0 fmol/mg of protein. Thus, high concentrations of PgR exist in benign liver tumors or hepatoblastoma, but not in HCC. At present, it is not known why most HCCs are negative for PgR or the titer is so low if present at all.

### *Operative Results of HCC*

In our series of 107 radical hepatic resections, the 1-, 3-, and 5-year survival rates in male and female patients were 78% and 70%, 45% and 52%, and 19% and 52%, respectively[38]. The difference was significant 47 months after operation. The clinicopathologic background was not different between men and women. Thereafter, we have analyzed the recurrence and survival rates between the patients with AR-negative and AR-positive HCCs[39]. The recurrence rates were 33.8% in the former and 67.9% in the latter group. Also, the 5-year survival rate was significantly better in the patients with AR-negative HCCs (62.2%) than in the patients with the positive tumors (17.3%). On the other hand, the presence or absence of ER in HCC did not influence the long term results[40].

### *Hormone Therapy of HCC*

Friedman *et al.*[22] first tried hormone therapy for HCC. Since in hormone-responsive tissue such as breast or endometrial carcinoma that contains ERs, administration of progestins results in decreased ER titers and often causes tumor regression, they performed progestin therapy in 5 patients with HCC. Tumor regression was observed in two of them. We have treated 9 patients with nonresectable HCC with tamoxifen, but no apparent tumor response was found (unpublished data). As tamoxifen inhibits the development of hyperplastic nodules in the liver of experimental animals[41], anti-estrogen therapy may be effective in selected patients with HCC. However, we probably cannot expect so much solely by anti-estrogen therapy in the treatment of far advanced HCCs. Based upon the fact that human HCCs contain AR, Forbes *et al.*[42] performed anti-androgen therapy with cyproterone acetate. They have found objective responses in 5 of 25 patients with advanced HCCs. We have also obtained a similar but a little worse result in patients with nonresectable or recurrent HCCs (unpublished data). Recently, Bannister *et al.*[43] and more recently Ostrowski *et al.*[44] reported that the development of HCC is strongly related to the increased hepatic AR concentrations in diethylnitrosamine induced hepatocarcinogenesis in rats. Using human HCC cell lines, Ohnishi *et al.*[45] have shown that the growth of AR positive cells was enhanced by dihydrotestosterone, and was inhibited by adding cyproterone acetate.

### *Future Perspective*

Except for oral contraceptive-associated HCCs which may be highly estrogen-dependent, human HCCs seem to be more androgen-dependent. Male predominance, the receptor profiles, and the clinical evidence may support this speculation. Routine receptor assays of surgically removed specimens will be useful for further elucidation of this unsolved problem. Based upon the receptor result in each individual, appropriate hormone therapy which may be anti-androgenic, anti-estrogenic, or both will be possible. The poor survival in men after liver resection could theoretically be improved by adjuvant anti-androgen therapy. It may be possible to improve the survival rate of those with nonresectable HCCs by combining hormone therapy with conventional chemo- or immuno-chemotherapy. If hormonal manipulation can prevent the development of HCC in high-risk patients will be a matter for future work.

### *References*

1. Van Thiel, D.H. and Lester, R. (1979) Hypothalamic-pituitary-gonadal dysfunction in patients with alcoholic liver disease. In: *Problems in Liver Disease*, ed, Davidson C.S. pp. 289–298. New York: Stratton Intercontinental
2. Nagasue, N., Ogawa, Y., Yukaya, H., Ohta, N. and Ito, A. (1985) Serum levels of estrogens and testosterone in cirrhotic men with and without hepatocellular carcinoma. *Gastroenterology*, **88**, 768–772
3. Baum, J.K., Bookstein, J.J., Holtz, F. and Klein, E.W. (1973) Possible association between benign hepatomas and oral contraceptives. *Lancet*, **2**, 926–929
4. Edmondson, H.A., Reynolds, T.B., Henderson, B. and Benton, B. (1977) Regression of liver cell adenoma associated with oral contraceptives. *Ann. Intern. Med.*, **86**, 180–182
5. Steinbrecher, U.P., Lisbona, R., Huang, S.N. and Mishkin, S. (1981) Complete regression of hepatocellular adenoma after withdrawal of oral contraceptives. *Dig. Dis. Sci.*, **26**, 1045–1050
6. Buhler, H., Pirovino, M., Akovbiantz, A. *et al.* (1982) Regression of liver cell adenoma: A follow-up study of three consecutive patients after discontinuation of oral contraceptive use. *Gastroenterology*, **82**, 775–782
7. Neuberger, J., Forman, D., Doll, R. and Williams, R. (1986) Oral contraceptives and hepatocellular carcinoma. *Br. Med. J.*, **298**, 1355–1357
8. Forman, D., Vincent, T.J. and Doll, R. (1986) Cancer of the liver and the use of oral contraceptives. *Br. Med. J.*, **292**, 1357–1361
9. Bernstein, M.S., Hunter, R.L. and Yachnin, S. (1971) Hepatomas and peliosis hepatis developing in a patient with Fanconi's anemia. *N. Engl. J. Med.*, **284**, 1135–1136
10. Lang, R. and Redmann, U. (1979) Nonmutagenicity of some sex hormones in the Ames Salmonella/microsome mutagenicity test. *Mutat. Res.*, **67**, 361–365

11. The Committee on Safety of Medicine (1972) Carcinogenicity tests of oral contraceptives. London: Her Majesty's Stationery Office
12. Reznik-Schuller, H. (1979) Carcinogenic effects of diethylstilbestrol in male Syrian golden hamsters and European hamsters. *J. Natl. Cancer Inst.*, **62**, 1083–1085
13. Taper, H.S. (1978) The effect of estradiol-17-phenylpropionate and estradiol benzoate on N-nitrosomorphine-induced liver carcinogenesis in ovariectomized female rats. *Cancer*, **42**, 462–467
14. Yager, J.D. and Yager, R. (1980) Oral contraceptive steroids as promoters of hepatocarcinogenesis in female Sprague-Dawley rats. *Cancer Res.*, **40**, 3680–3685
15. Wanless, I.R. and Medline, A. (1982) Role of estrogens as promoters of hepatic neoplasia. *Lab. Invest.*, **46**, 313–320
16. Sumi, C., Yokoro, K., Kajitani, T. and Ito, A. (1980) Synergism of diethylstilbestrol and other carcinogens in concurrent development of hepatic, mammary, and pituitary tumors in castrated male rats. *J. Natl. Cancer Inst.*, **65**, 169–175
17. Sumi, C., Yokoro, K. and Matsushima, R. (1983) Induction of hepatic tumors by diethylstilbestrol alone or in synergism with N-nitrosobutylurea in castrated male WF rats. *J. Natl. Cancer Inst.*, **70**, 937–942
18. Higashi, S., Tomita, T., Mizumoto, R. and Nakakuki, K. (1980) Development of hepatoma in rats following oral administration of synthetic estrogen and progestogen. *Gann*, **71**, 576–577
19. Althouse, R., Huff, J., Tomatis, L. and Wilbourn, J. (1980) An evaluation of chemicals and industrial processes associated with cancer in humans based on human and animal data: IARC monographs volumes 1 to 20. *Cancer Res.*, **40**, 1–12
20. Morris, H.P. and Firminger, H.I. (1956) Influence of sex and sex hormones on development of hepatomas and other hepatic lesions in Starin AxC rats ingesting 2-diacetylaminogluorene. *J. Natl. Cancer Inst.*, **16**, 927–949
21. Duffy, M.J. and Duffy, G.J. (1978) Estradiol receptors in human liver. *J. Steroid Biochem.*, **9**, 233–235
22. Friedman, M.A., Demanes, D.J. and Hoffman, P.G. Jr. (1982) Hepatomas: Hormone receptors and therapy. *Am. J. Med.*, **73**, 362–366
23. Nagasue, N., Ito, A., Yukaya, H. and Ogawa, Y. (1986) Estrogen receptors in hepatocellular carcinoma. *Cancer*, **57**, 87–91
24. Nagasue, N., Kohno, H., Chang, Y-C. *et al.* (1989) Androgen and estrogen receptors in hepatocellular carcinoma and the surrounding liver in women. *Cancer*, **63**, 112–116
25. Ohnishi, S., Murakami, T., Moriyama, T., Mitamura, K. and Imawari, M. (1986) Androgen and estrogen receptors in hepatocellular carcinoma and the surrounding noncancerous liver tissue. *Hepatology*, **6**, 440–443
26. Li, S.A. and Li, J. (1981) Changes in estrogen receptor levels during DES-induced hepatocarcinogenesis in the Syrian hamster fed α-naphtoflavon. *J. Steroid Biochem.*, **15**, 387–392
27. Nagasue, N., Ito, A., Yukaya, H. and Ogawa, Y. (1985) Androgen receptors in hepatocellular carcinoma and surrounding parenchyma. *Gastroenterology*, **89**, 643–647
28. Bannister, P., Sheridan, P. and Losowsky, M.S. (1985) Identification and characterization of the human hepatic androgen receptor. *Clin. Endocrinol.*, **23**, 495–502
29. Iqbal, M.J., Wilkinson, M.L., Johnson, P.J. and Williams, R. (1983) Sex steroid receptor protein in foetal, adult and malignant human liver tissue. *Br. J. Cancer*, **48**, 791–796
30. Nagasue, N., Yukaya, H., Chang, Y-C., Ogawa, Y., Kohno, H. and Ito, A. (1986) Active uptake of testosterone by androgen receptors of hepatocellular carcinoma in humans. *Cancer*, **57**, 2162–2167
31. Nagasue, N., Yukaya, H., Hamada, T., Hirose, S., Kanashima, R. and Inokuchi, K. (1984) The natural history of hepatocellular carcinoma: a study of 100 untreated cases. *Cancer*, **54**, 1461–1465
32. Peters, R.I. (1976) Pathology of hepatocellular carcinoma. In: Okuda, K., Peters, R.I., eds., *Hepatocellular Carcinoma*. John Wiley & Sons, New York, pp. 107–168
33. MacDonald, J.S., Lippman, M.E., Woolley, P.V., Petrucci, P.P. and Schein, P.S. (1978) Hepatic estrogen and progesterone receptors in an estrogen-associated hepatic neoplasm. *Cancer Chemother. Pharmacol.*, **1**, 135–138
34. Bojar, H., Petzinna, D., Brolsch, Ch. and Staib, W. (1984) Steroid receptor status of focal-nodular hyperplasia of the human liver. *Klin. Wochenschr.*, **62**, 446–450
35. Carbone, A. and Vecchio, F.M. (1986) Presence of cytoplasmic progesterone receptors in hepatic adenomas: A report of two cases. *Am. J. Clin. Pathol.*, **85**, 325–329
36. Demenes, D.J., Friedman, M.A., McKerrow, J.H. and Hoffman, P.G. (1982) Hormone receptors in hepatoblastoma: A demonstration of both estrogen and progesterone receptors. *Cancer*, **50**, 1828–1832
37. Nagasue, N., Kohno, H., Yamanoi, A., Kimoto, T., Chang, Y-C. and Nakamura, T. (1991) Progesterone receptor in hepatocellular carcinoma: Correlation with androgen and estrogen receptors. *Cancer*, **67**, 2501–2505
38. Nagasue, N., Galizia, G., Yukaya, H., Kohno, H., Chang, Y-C., Hayashi, T. and Nakamura, T. (1989) Better survival in women than in men after radical resection of hepatocellular carcinoma. *Hepato-Gastroenterol.*, **36**, 379–383
39. Nagasue, N., Chang, Y-C., Hayashi, T. *et al.* (1989) Androgen receptor in hepatocellular carcinoma as a prognostic factor after hepatic resection. *Ann. Surg.*, **209**, 424–427
40. Nagasue, N., Kohno, H., Chang, Y-C., *et al.* (1990) Clinicopathologic comparisons between estrogen receptor-positive and -negative hepatocellular carcinomas. *Ann. Surg.*, **212**, 150–154
41. Kohigashi, K., Fukuda, Y. and Imura, H. (1988) Inhibitory effect of tamoxifen on diethylstilbestrol-promoted hepatic tumorigenesis in male rats and its possible mechanism of action. *Jpn. J. Cancer Res.*, **79**, 1335–1339
42. Forbes, A., Wilkinson, M.L., Iqbal, M.J., Johnson, P.J. and Williams, R. (1987) Response to cyproterone acetate treatment in primary hepatocellular carcinoma is related to fall in free 5$\alpha$-dihydrotestosterone. *Eur. J. Cancer Clin. Oncol.*, **23**, 1659–1664
43. Bannister, P., Parsons, M.A., Ingleton, P., Underwood, J.C.E. and Losowsky, M.S. (1986) Androgen receptor concentrations in the diethylnitrosamine model of hepatic carcinogenesis. *Br. J. Cancer*, **54**, 857–859
44. Ostrowski, J.L., Ingleton, P.M., Underwood, J.C.E. and Parsons, M.A. (1988) Increased hepatic androgen receptor expression in female rats during diethylnitrosamine liver carcinogenesis: A possible correlation with liver tumor development. *Gastroenterology*, **94**, 1193–1200
45. Ohnishi, S., Ishikawa, T., Matsuhashi, N. *et al.* (1987) The effects of testosterone and anti-androgen administrations on the human hepatoma cell lines with androgen receptors. *Acta Hepatol. Jap.*, **28**, 178

*(On invitation by S. Bengmark 3 February 1992)*

